# Protocol to examine the neural basis of symbolic and non-symbolic quantity processing in human brain with fMRI

**DOI:** 10.1016/j.xpro.2022.101673

**Published:** 2022-09-13

**Authors:** Simge Altınok, Gözde Vatansever, Sertaç Üstün, Emre H. Kale, Metehan Çiçek

**Affiliations:** 1 Department of Interdisciplinary Neuroscience, Ankara University, Ankara, Turkey; 2 Brain Research Center, Ankara University, Ankara, Turkey; 3 Neuroscience and Neurotechnology Center of Excellence (NÖROM), Ankara, Turkey; 4 Department of Physiology, School of Medicine, Ankara University, Ankara, Turkey

**Keywords:** Clinical Protocol, Neuroscience, Cognitive Neuroscience, Behavior

## Abstract

Number perception is among the basic cognitive abilities necessary to understand our environment. Here, we present a protocol to examine the neural underpinnings of numerosity comparison regarding symbolic and non-symbolic stimuli using functional magnetic resonance imaging (fMRI). This protocol gives instructions for screening participants, followed by steps to perform an event-related fMRI experiment and data analysis with SPM12. This protocol will be informative for investigating numerical cognition in various groups including children with dyscalculia or people at different developmental stages.

For complete details on the use and execution of this protocol, please refer to Üstün et al. (2021) and Vatansever et al. (2020).

## Highlights

• An fMRI protocol to study neural basis of quantity processing at varied difficulty levels
• Applicable to study numerosity in dyscalculia and at varied developmental stages
• Instructions on how to conduct an event-related fMRI study
• Guidelines to perform a first-level and second-level fMRI data analysis using SPM12

## Abstract (editor highlights)

Publisher’s note: Undertaking any experimental protocol requires adherence to local institutional guidelines for laboratory safety and ethics.

## Graphical abstract

**Figure:**
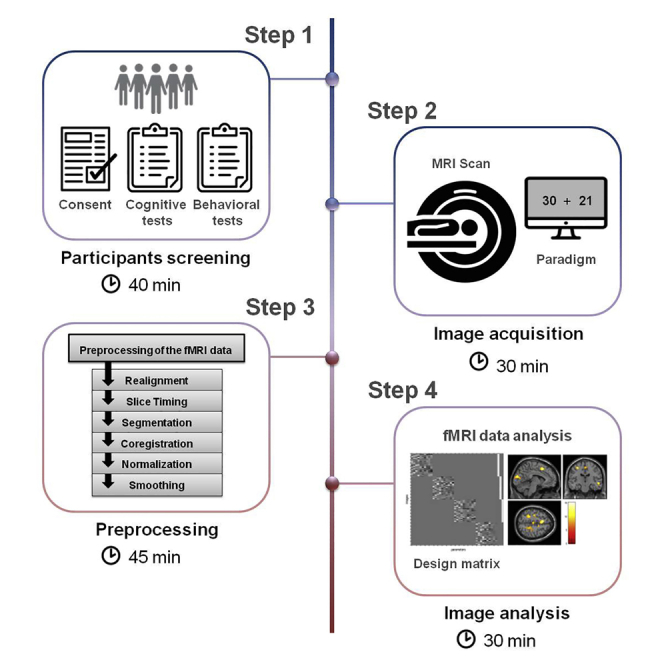

## Before you begin

The protocol below describes the instructional steps to examine neural underpinnings of numerosity comparison at various difficulty levels (easy and difficult) using fMRI.

### Institutional permissions

All scientific research on human participants must be approved by a local ethical committee before beginning. This protocol is derived from our numerical cognition studies involving human participants (Vatansever et al., 2020; Üstün et al., 2021). The research method used in these studies are approved by The Ethical Board of the Medical Faculty of Ankara University.

### Preparing the experimental materials

**Timing: flexible (preliminary works)**

**Figure 1:**
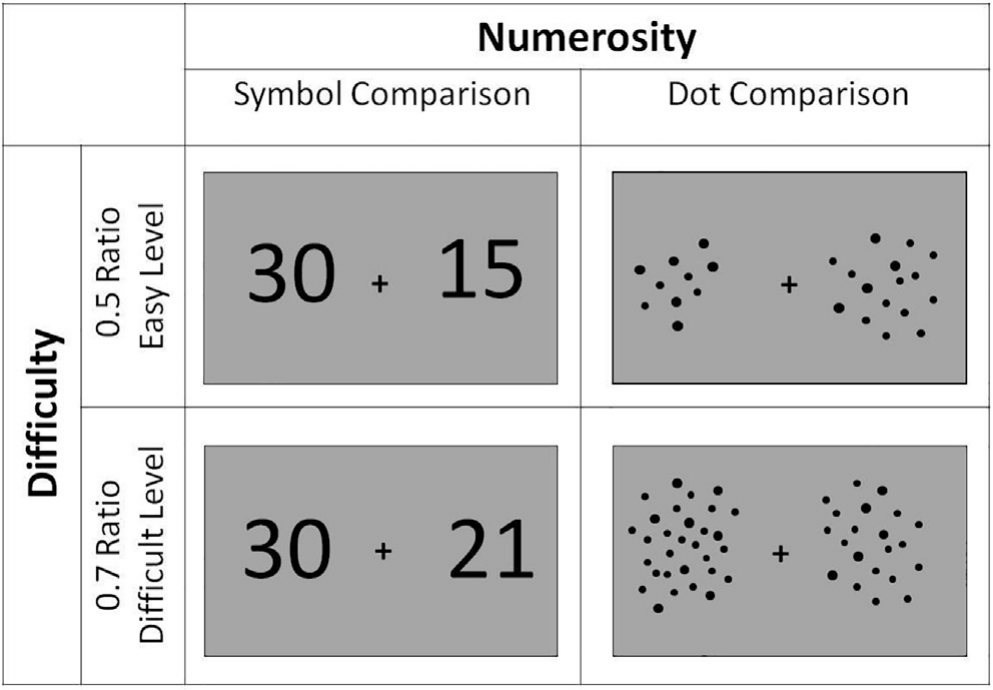
Summary of numerosity and difficulty conditions Figure adapted with permission from (Üstün et al., 2021).

1. Design a 2 × 2 numerosity comparison task. Prepare visual images according to two numerosity conditions at two difficulty levels based on the following principles (Figure 1):
  a. Arabic numbers (symbolic condition).
    i. During pseudo-randomized presentation, avoid the Arabic numbers that have shared visual similarities.
    ii. Use a 300-point font for numbers in the visual stimuli so as not to allow the size of symbols to interfere with the quantity comparison.
      ***Note:*** In symbolic condition, a pair of double-digit Arabic numbers are presented on the left and right sides of the gray screen.
  b. The arrays of dots (non-symbolic condition).
    i. Determine dot arrays to equate visual properties in all stimuli.
    ii. Change the size of dots, as well as density and cumulative surface area of dot arrays across trials to prevent participants from attending to spatial features of stimuli rather than the numerical.
      ***Note:*** In non-symbolic condition, arrays of black dots in different quantities are presented in both sides of the gray screen.
      ***Note:*** In half of the trials, the surface area is greater for the larger value, and in contrast, in the other half for the smaller value.
      ***Note:*** The difficulty conditions of the task are manipulated by the quantity ratios between the comparison pairs of visual stimuli for symbolic and non-symbolic conditions. Thus, the distance between the pair of numerosities of the easy level can be arranged to be further apart than the difficult level.
  c. Determine the two difficulty levels, easy and difficult, for both task conditions.
    i. Arrange pairs of quantities in both symbolic and non-symbolic task conditions with a distance ratio of 0.6 on the easy level.
    ii. Set the two numerosities for both task conditions with a distance ratio of 0.8 on the difficult level.
      **CRITICAL:** To equalize the performance among participants of different ages, different difficulty levels should be chosen by considering the behavioral performance differences. The difficulty levels given above are used for our adult study (Vatansever et al., 2020). The most appropriate way to determine difficulty levels may be to conduct pilot behavioral experiments. For instance, in our study involving 3rd-grade children with math learning difficulties (i.e., dyscalculia), the difficulty levels were determined as 0.5 for the easy level and 0.7 for the difficult level according to the pilot-study findings (Üstün et al., 2021). In order to avoid ceiling and floor effects, we determined difficulty levels according to the percentage of correct responses of the participants in the pilot study.
      ***Note:*** The set of stimuli consisting of pairs of quantities is pre-determined for each ratio, and the same set is used for both symbolic and non-symbolic numerosity conditions. All these quantities correspond to double-digit numbers in symbolic condition and the arrays of dots in non-symbolic condition.
      ***Note:*** Since the most frequently used paradigms in the literature are based on the visual modality, we also used the visual task paradigm. However, it would be valuable to examine the cross-modal abilities during the evaluation of the numerosity comparison responses.

### Participants screening

**Timing: 40 or 60 min per participant (depending on the inclusion of mock scanner training)**

2. Have participants complete a written consent form to ensure that they are informed about the content of the study and any risks or benefits associated with it (∼ 5 min).
3. Have participants complete a demographic form (including age, education level, gender, history of neurological and/or psychiatric diseases, medication, etc.) (∼ 5 min).
  a. Right-hand preference and normal fluid intelligence score are expected for all participants.
  b. Determine fluid intelligence scores by The Raven Progressive Matrices tests norms (∼ 20 min).
  c. Evaluate the mathematical abilities to control differences between study groups (∼ 5 min).
4. After psychometric evaluations (such as hand preference, fluid intelligence and mathematical abilities) are completed, proceed to the MR scanning for adults.

**CRITICAL:** Alternatively, in experiments including under-age participants (or adults who are hesitant about MRI scanning), subjects should be familiarized with the MRI procedure beforehand with a training session inside a mock scanner. This stage would be useful to minimize head movement artifacts and anxiety during the real MR scanning session (∼ 15 min). Troubleshooting 1.

5. For those who got a mock scanner training, arrange another suitable day for the fMRI scan (∼ 5 min).

**CRITICAL:** It is strongly recommended to conduct a pilot study before the experiment and run a power analysis with these data to estimate the optimum number of participants.

***Note:*** If you have under-age participants, a screening for children with math learning difficulties (i.e., dyscalculia) is important. In our study, which included 3rd-grade children in the Turkish sample, dyscalculia was evaluated as having mathematical skills at the lowest 25th percentile (Üstün et al., 2021). Also, having comorbid disorders including attention deficit hyperactivity disorder (ADHD) and anxiety disorder, having low reading ability denoting dyslexia, and an overall IQ score under 80 are defined as exclusion criteria for children in our study. These criteria for defining dyscalculia may vary in different cultures or populations but will be important for adapting this protocol to studies involving children.

### MRI scanner setup

**Timing: 15 min per participant**

6. Turn on the stimulus computer and check that the monitor resolution and refresh rate are set properly. In our experiment, the tasks are presented on a 28 × 37.5 cm screen with 72.5 cm from the participant’s eyes to the screen. The monitor resolution is 1024 × 768 pixels, and the refresh rate is 60 Hz.
7. Set the projection screen or monitor behind the MR machine based on the viewing distance.
8. Before entering the scanner room, ask participants to remove any metal-containing objects. To reduce the impact of noise of the scanner, give earplugs to the participant and show them how to put the earplugs on.
9. Set cushions for the head and legs on the MR table and ask the participant to lie in a comfortable position.
10. Place the MR-compatible response pad under the participant’s right hand. Let them place their fingers on the buttons and try.

**CRITICAL:** Ensure that MRI compatible equipment, such as the response pad, is working properly before beginning data collection. Troubleshooting 2.

11. Adjust and plug the head coil. Let the notches on the head coil be at the level of the eyebrows, and the middle of the coil divides the face in half.

***Note:*** If possible, use the laser marking system for better positioning of participant’s head.

***Note:*** Participant’s eyes must be closed while using laser marking. The laser marking should be in the middle of the face at the level of the notches and eyebrows.

***Note:*** This process pre-determines the optimum position of the participant’s head. Thus, when the MRI table moved inside the magnet, the participant’s head would be on the isocenter, making the image acquisition phase as identical as possible between subjects.

12. Stabilize the participant’s head inside the head coil using cushions.
13. Place the mirror on the head coil allowing participants to see visual stimuli on the MRI-compatible screen.
14. Move the MR table into the scanner bore.
15. Ask the participant if they can see all parts of the projection screen clearly and adjust it as required.
16. Tell the participant to use the squeeze ball if they feel uncomfortable inside the scanner.
17. Exit the scanner room and get onto the stimulus computer.
18. Talk to participants via intercom unit and make sure they can hear your voice.

## Key resources table

**Table:** 

REAGENT or RESOURCE	SOURCE	IDENTIFIER
**Deposited data**		

Psychtoolbox code to run numerosity comparison paradigm used in female and male children aged between 10-13 (easy level with 0.5 and difficult level with 0.7 ratio)	Üstün et al. (2021)	Open Science Framework (https://osf.io/b85xg/) with the identifier https://doi.org/10.17605/OSF.IO/B85XG
Psychtoolbox code to run numerosity comparison paradigm used in female and male adults aged between 20-23 (easy level with 0.6 and difficult level with 0.8 ratio)	Vatansever et al. (2020)	Open Science Framework (https://osf.io/b85xg/) with the identifier https://doi.org/10.17605/OSF.IO/B85XG

**Experimental models: Organisms/strains**		

Human participants (31 Turkish participants; 16 healthy young adults, age range: 20–23, 9 female; 15 healthy children, age range: 10–13, 8 female)	Vatansever et al. (2020)	N/A

**Software and algorithms**		

MATLAB R2017a	https://www.mathworks.com/	RRID: SCR_001622
Psychtoolbox v3.0.13	http://psychtoolbox.org/	RRID: SCR_002881
SPM12	https://www.fil.ion.ucl.ac.uk/spm/	RRID: SCR_007037
IBM SPSS Statistics 23.0	https://www.ibm.com/products/spss-statistics	RRID: SCR_019096
Monte Carlo simulation	Slotnick (2017)	https://www.fil.ion.ucl.ac.uk/spm/
MotionFingerprint toolbox (if your study sample includes children)	Wilke (2012)	https://doi.org/10.1016/j.neuroimage.2011.10.043
MarsBaR toolbox	Brett et al. (2002)	https://marsbar-toolbox.github.io/index.html
EPI template (SPM)	Talairach and Tournoux (1988)	Montreal Neurological Institute (MNI)
Template-O-Matic Toolbox (if your study sample includes children)	Wilke et al. (2008)	http://www.neuro.uni-jena.de/software/ tom

**Other**		

3-T Siemens Magnetom Trio MRI system with a 32-channel head-coil array	UMRAM - National Magnetic Resonance Research Center, Bilkent University	https://www.siemens-healthineers.com/en-us/refurbished-systems-medical-imaging-and-therapy/ecoline-refurbished-systems/magnetic-resoncance-imaging-ecoline/magnetom-trio-3t-eco

## Step-by-step method details

### Numerosity comparison task

**Timing: Approximately 15 min per participant**

In this section, we described the instructions for applying the experimental paradigm. Furthermore, we mentioned the technical prerequisites that should be checked before starting the experiment. Finally, we highlighted the key points of the experimental design for the neuroimaging phase.

1. Before starting the experiment, make sure the PC that you run the experimental task and the screen system of the scanner are connected.
2. The task is displayed on the PC running the Psychtoolbox code via MATLAB. Participants see the visual images on the MRI-compatible screen via a mirror placed in the head-coil. Troubleshooting 3.

**CRITICAL:** If participant cannot see the task due to a visual impairment, use MR-compatible glasses to correct refractive errors.

**Figure 2:**
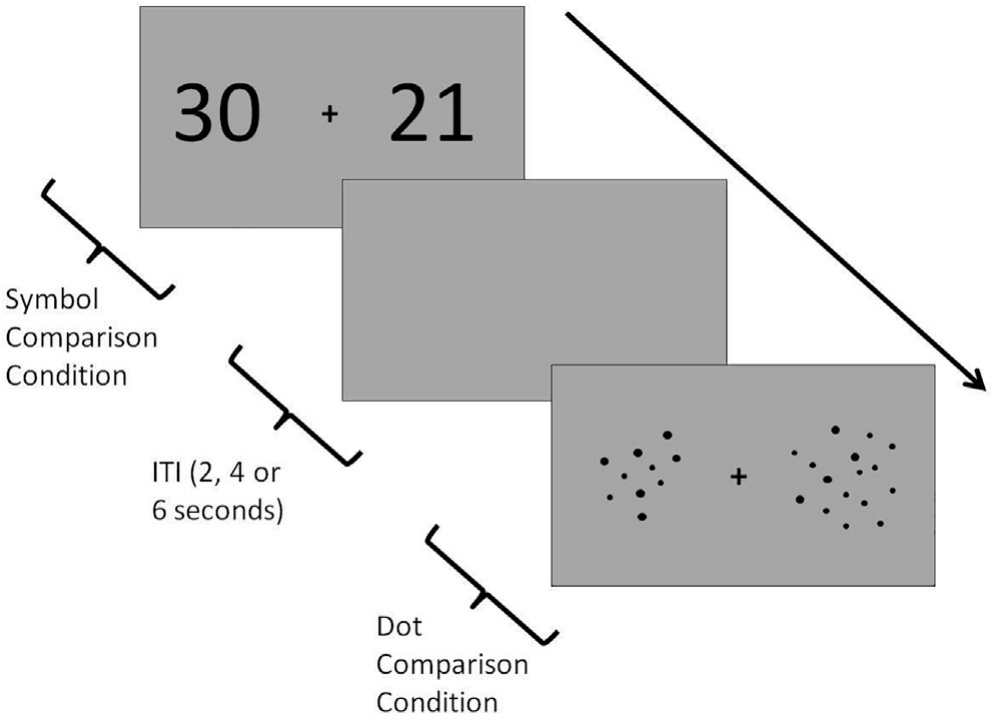
Numerosity comparison paradigm depicting non-symbolic and symbolic conditions Figure adapted with permission from (Vatansever et al., 2020).
4. Design an experiment with four scan runs, each lasting approximately 3 min 20 s.
  a. Set every experimental run to include 36 trials.
  b. Set all trials as 2 s long.

***Note:*** Each experimental run consists of 9 trials of each condition (symbolic and non-symbolic) as well as each difficulty level (easy and difficult) pair.

3. Give the following instructions to the participants for the numerosity comparison paradigm (Figure 2):
  a. You will see a pair of stimuli composed of dot arrays or Arabic numbers which are presented simultaneously on the left and right sides of the gray screen throughout the numerosity comparison task.
  b. Select the larger quantity in each pair of stimuli by pressing the button corresponding to the side on the screen.

5. Present four different trial types in a randomized order. Set the inter-trial intervals (with the presentation of a fixation cross in the center of the screen) to 2, 4, and 6 s arranged in a pseudo-randomized and logarithmic manner favoring shorter durations.
6. Train participants for behavioral paradigm by a computer before going into the scanner.

**CRITICAL:** The training session should be long enough for participant fully understand the task but not so long as to make them too familiar with it.

### Image acquisition

**Timing: Approximately 30 min per participant**

Throughout the neuroimaging session, participants lie down in the supine position in the scanner. Participants perform the numerosity comparison task during the fMRI scan. All responses are collected with a response pad positioned under participants’ right hands. The purpose of the neuroimaging session is to detect the brain activity related to symbolic and non-symbolic quantity processing. Troubleshooting 4.

***Note:*** In our original study, we used a 3-T Siemens Magnetom Trio MRI system with a 32-channel head-coil array to obtain fMRI images.

7. Following instructions reveal how to start the scanning session.
  a. Register the participant to the computer system of the scanner.
  b. Open the experiment protocol.
  c. Inform the participant that localizer acquisition is about the start and remind not to move.
    **CRITICAL:** Check the localizer acquisition in the computer screen and make sure the participant's head is adjusted on the alignment of the isocenter. If there are any metallic objects left on the participant, the image will reveal defects caused by magnetic area disturbances.
  d. Image should be seen in three main axes (coronal, sagittal and horizontal) on the computer screen.
  e. Before starting the anatomical acquisition, make sure that the slice placement is adjusted to cover the entire brain.
  f. Acquire anatomical images using T1-weighted sequence (Time to Repeat (TR): 2,600 ms, Time to Echo (TE): 3.02 ms, Field of View (FOV): 256 mm, matrix: 256 × 256 and slice thickness: 1.00 mm).
  g. Start the experimental paradigm.
  h. Acquire functional image using 46 slices with 3-mm width and a 0-mm gap (TR: 2,600 ms, TE: 28 ms, matrix: 64 × 64, FOV: 192 mm, voxel size: 3 × 3 × 3 mm).
  i. Repeat the functional acquisition to achieve four experimental runs.
  j. Save all data obtained in the experiment.
  k. Finish the session by moving the bed out of the scanner and slowly getting the participant up.

## Expected outcomes

After completing the experiment, anatomical and functional data will be obtained for each participant. In this protocol, we collect brain images through four functional runs. Each functional run consists of 73 TRs (or images), thus 292 functional images in total are acquired for one participant. The functional data is used to understand brain activity underlying symbolic and non-symbolic quantity processing, while the anatomical data is used in the preprocessing stage of the fMRI data.

## Quantification and statistical analysis

Brain images can be analyzed with different kinds of tools or software. Furthermore, applications for these analyses may vary between researchers as well as among neuroimaging laboratories. To gain a full and better perspective about these practices of the neuroimaging analysis, you may check Nichols et al. (2017). Here, we share our approach to analyze the neural activations rather than a standard guideline for neuroimaging analysis. In this paper you will find a pipeline of analysis that is used in our fMRI studies of numerical cognition in children with dyscalculia (Üstün et al., 2021) and people at different developmental stages (Vatansever et al., 2020). In the scope of this protocol, the event-related functional images will be processed using SPM12 software, which needs MATLAB software to run.

### Behavioral analysis

**Timing: Approximately 10 min for a computer (64 bit, 16 GB RAM, Intel(R) Xeon(R) CPU E5-1650 v4 @ 3.60GHz 3.60 GHz)**

1. After the fMRI session, you will get two behavioral parameters; the response time and the percentage of correct responses that are the output of paradigm codes.
2. The behavioral data is separately analyzed using repeated measures of ANOVA with the factors of numerosity, difficulty, and group to reveal potential interactions between the factors.

### Converting raw fMRI data to .img extension format

**Timing: Approximately 5 min per participant for a computer (64 bit, 16 GB RAM, Intel(R) Xeon(R) CPU E5-1650 v4 @ 3.60GHz 3.60 GHz)**

**Figure 3:**
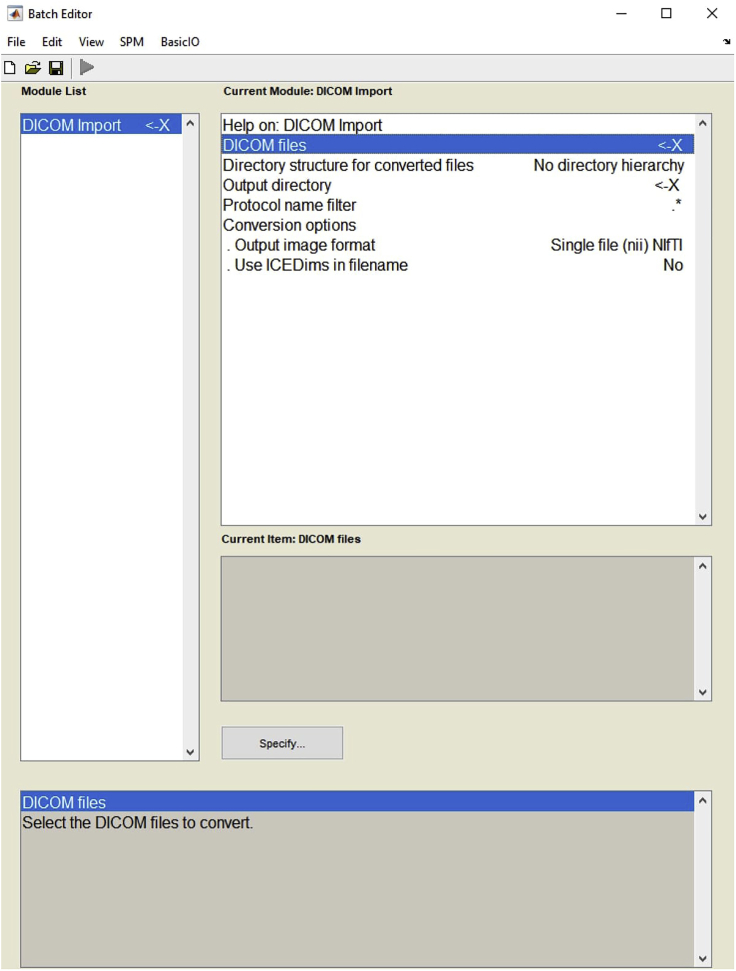
Example view of the batch editor for DICOM import

3. Open MATLAB and set the folder that contains imaging data as the main directory.
4. To convert DICOM images to NIFTI file format, launch the SPM toolbox on MATLAB. Click the DICOM import on the SPM12 menu and upload DICOM files (Figure 3). Use different folders to define as output directory for the data obtained: One for anatomical and four other for functional runs.

### Preprocessing of the fMRI data

**Timing: Approximately 45 min per participant for a computer (64 bit, 16 GB RAM, Intel(R) Xeon(R) CPU E5-1650 v4 @ 3.60GHz 3.60 GHz)**

5. Remove the first five images of each run by deleting the corresponding files due to the initial irregularity of the MR signal and do not include these files to the analysis.
6. On the SPM12 menu, click on the Batch editor. Use the upper menu to add each preprocessing step you need. On the batch editor, for all the preprocessing steps, you should load the image files sequentially (Figure 4).

**Figure 4:**
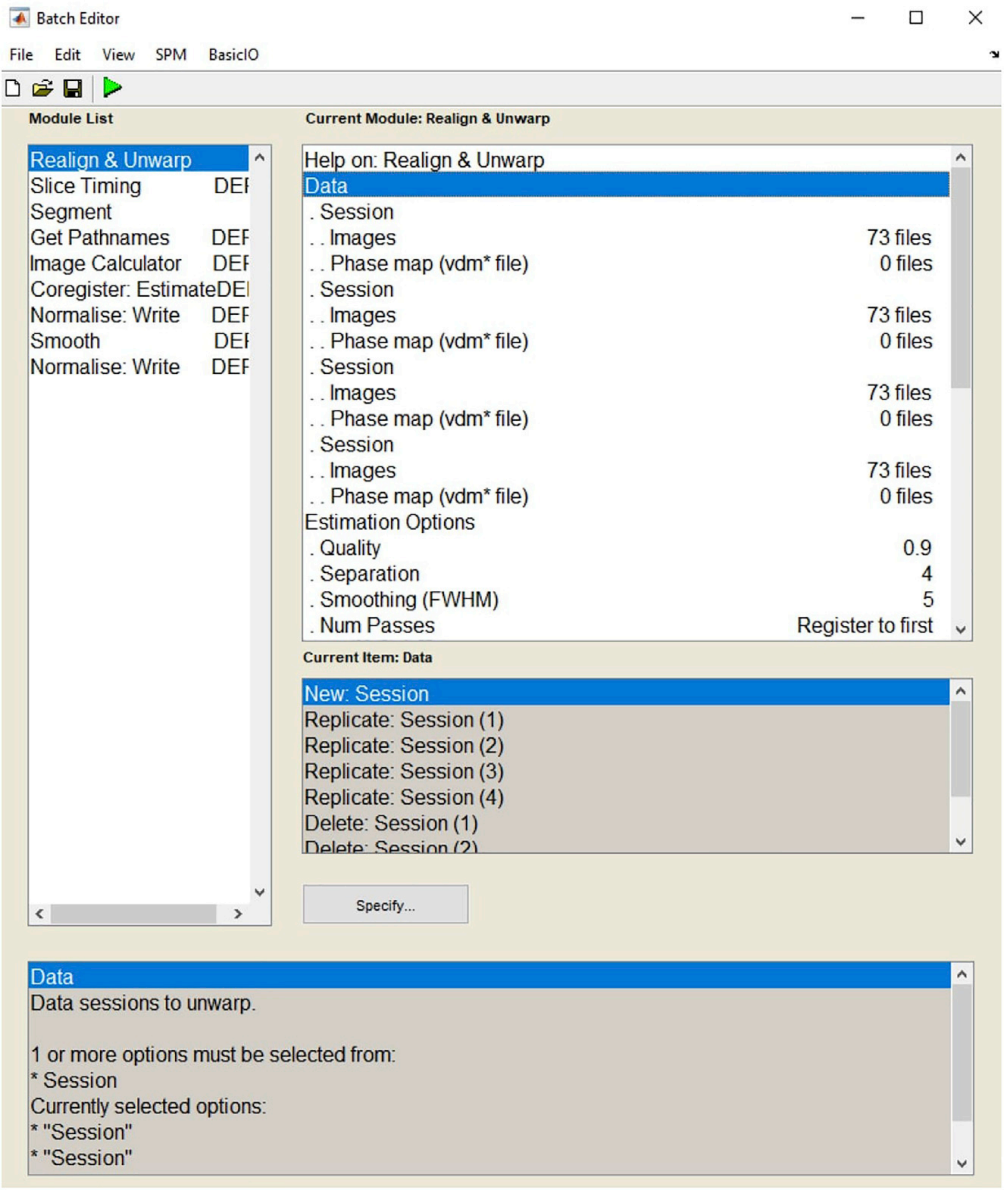
Example view of the batch editor for preprocessing
7. Apply the preprocessing stages to fMRI data.
  a. Realignment: The functional images are realigned to correct for the movement artifacts.
    i. There should be four sessions corresponding to each functional run. To add a new session, click on the “Data” and then the “New: Session”. If you open any redundant session, use “Delete: Session” to delete it.
  b. Slice timing correction: This step shifts the time series of each voxel relative to the template (usually the middle slice) so they all appear to be sampled at the same time. In slice timing, define the existing sections using the scanning parameters of your study (check the below parameters with the technician in your MRI center). But as an example, the parameters were as follows for the MR sequence used in our original study:
    i. Define the Number of Slices as 46.
    ii. Define the TR as 2600.
    iii. Define the TA value. The TA value is calculated by SPM based on the information provided. It is usually calculated as *TR-(TR/nslices).*
    iv. Fill in the “Slice Order” section with exact sequence of the acquisition of the 46 slices.
    v. Select the Reference Slice as 1.
  c. Segmentation: The segmentation allows defining three tissue types including the gray matter, the white matter, and the cerebrospinal fluid as separately.
    i. Upload the anatomical image on the Volumes under the Data section.
    ii. Under the Custom section, the Tissue Probability Maps should be defined in the order as Grey, White, and Cerebrospinal Fluid separately using three file directories’ names:
      C:\MatlabToolbox\spm12\tpm\gray.nii,1.
      C:\MatlabToolbox\spm12\tpm\white.nii,1.
      C:\MatlabToolbox\spm12\tpm\csf.nii,1.
    iii. In addition to three tissue types, SPM12 also allows defining the skull, the scalp, and the air in the segmentation. Enter them as follows:
      C:\MatlabToolbox\spm12\tpm\skull.nii,1.
      C:\MatlabToolbox\spm12\tpm\scalp.nii,1.
      C:\MatlabToolbox\spm12\tpm\air.nii,1.
      **CRITICAL:** The aforementioned names of files are used for explanation only. You can define all six maps by using tpm.nii file. Always check the file addresses. If working directory is not compatible with the batch and file directories on the computer, the batch will not work.
  d. Co-registration: The realigned functional images are co-registered with high-resolution anatomical T1 images to enable anatomical localization.
    i. The automatically selected mean image is used as the “Reference Image”.
    ii. Upload the converted T1 image as the “Source Image”.
  e. Spatial normalization: The anatomical and functional images are spatially normalized to MNI coordinates using the EPI template (Talairach and Tournoux, 1988).
    **CRITICAL:** In experiments involving children, it would be convenient to use a special template generated for a participant's mean age by the TOM toolbox (Template-O-Matic toolbox) to overcome normalization errors caused by differences in brain sizes (Wilke et al., 2008).
  f. Smoothing: Apply spatial smoothing using 9-mm full-width half-maximum Gaussian kernel.
    ***Note:*** In addition to preprocessing, the MotionFingerprint toolbox (Wilke, 2012) can be used to detect participants' head movement artifacts, especially in studies including children. It provides two head movement parameters for each volume, total displacement and scan-to-scan displacement. These parameters can be analyzed using the SPSS software to detect group differences regarding head movement artifacts. If there was a significant difference in the groups' head movement parameters, it would be useful to add them as regressors in the general linear model (GLM) to control. Also, data from participants with extreme head movement artifacts could be excluded from the study. Troubleshooting 5.

### Analysis of the fMRI data

**Timing: Approximately 30 min per participant for a computer (64 bit, 16 GB RAM, Intel(R) Xeon(R) CPU E5-1650 v4 @ 3.60GHz 3.60 GHz)**

8. The fMRI data is analyzed using SPM12 software, which needs MATLAB software to run.
9. The general linear model is used to perform the first-level analysis of the participants’ brain images. According to this protocol, the design matrix of the GLM would be constructed for every session by defining four conditions (symbolic-easy, symbolic-difficult, non-symbolic-easy, non-symbolic-difficult) and six motion parameters as regressors (also a session mean is calculated by the software). After the beta values are estimated considering the average of the four sessions, the contrast images can be created for different conditions.
10. The first-level analysis of the fMRI data is executed one by one for all participants. Once you load the batch, you can get the code for it from the batch editor (*View > Show .m Code*) and automatize these steps to work on every participant by editing the code (Figure 5).

**Figure 5:**
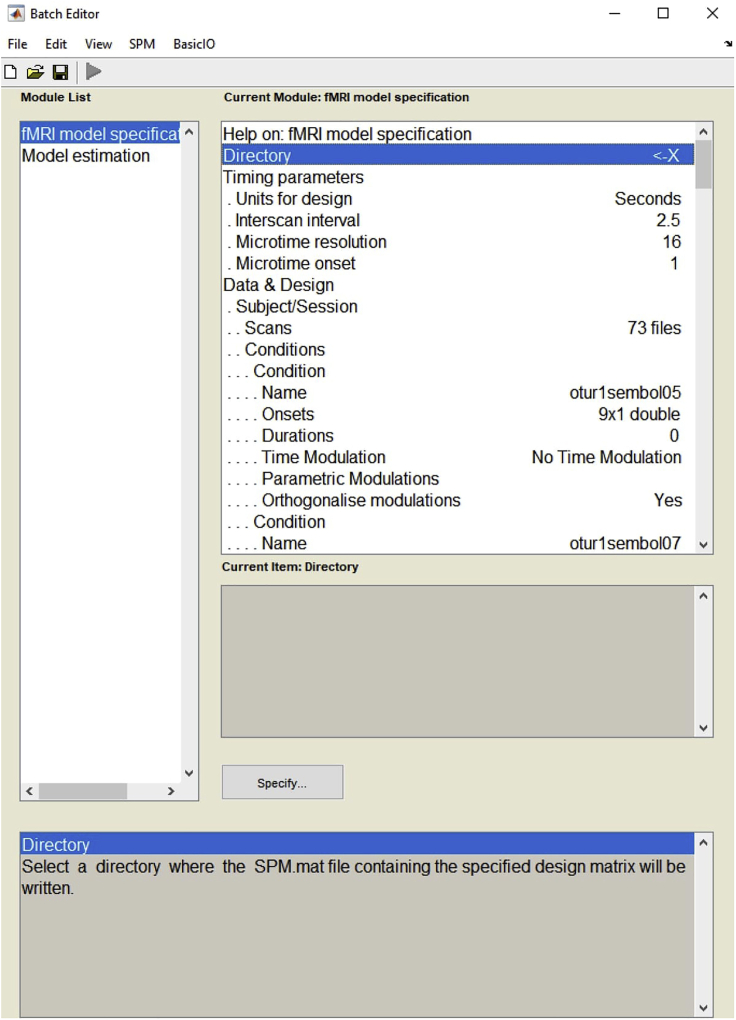
Example view of the batch editor for first-level analysis
11. Apply the first-level analysis steps to the fMRI data.
  a. Click on the Specify 1st-level section on the SPM menu.
  b. Enter the address of the output folder to the Directory.
  c. Under the Timing parameters section, enter the scanning parameters.
  d. Under the Data & Design section select “Scans”, to load the fMRI data of the first experimental run that has gone through all the preprocessing steps.
  e. Define four conditions as symbolic-easy, symbolic-difficult, non-symbolic-easy and non-symbolic-difficult respectively in the Condition section.
  f. Under the Condition section, enter the trial onsets of that condition into the Onsets section.
  g. Lastly, load the head movement parameters of the first experimental run into the Multiple regressors section.
  h. Define the high-pass filter value. The default cut-off is 128 s.
  i. To apply all these steps to fMRI data achieved from each experimental run, complete the sections under the Scans in an identical way for the other three runs.
  j. Under the Factorial Design Section, define the name of the factors along with the number of levels. Two factors with two levels for this protocol.
12. In this protocol, the four conditions for each experimental run, symbolic-easy, symbolic-difficult, non-symbolic-easy, and non-symbolic-difficult are the regressors of interest in the GLM.
13. The six-movement parameters obtained from the participants (in the realignment stage of the preprocessing) are included as regressors of no interest in the design matrix of the model (Figure 6).

**Figure 6:**
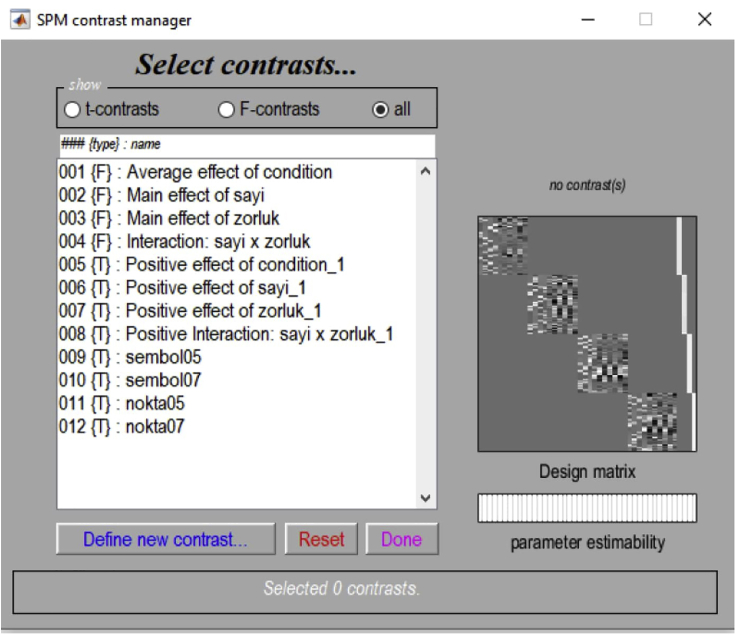
Example view of the design matrix
14. The second-level analysis of the fMRI data is run by three-way repeated-measures analysis of variance (ANOVA) using the SPM12 flexible factorial design feature (Figure 7).

**Figure 7:**
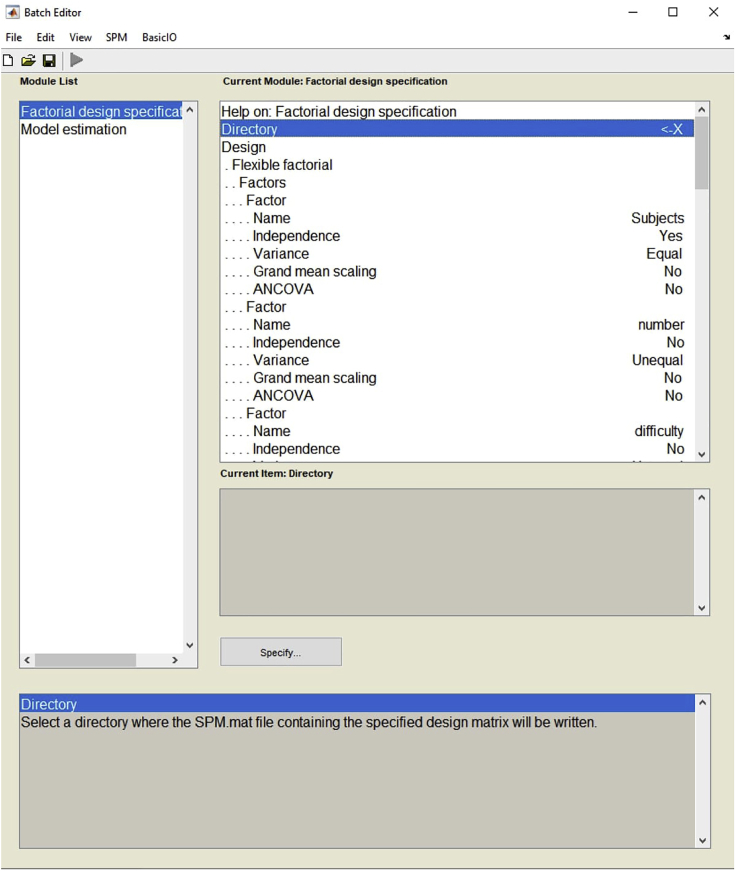
Example view of the batch editor for second-level analysis
15. The three factors, each with two levels, composed of (number: symbolic / non-symbolic) × (difficulty: easy / difficult) × (group: group 1 / group 2) are involved in the ANOVA analysis.

**CRITICAL:** The fact that this protocol is based on our studies examining neural differences in quantity processing between the two groups (Vatansever et al., 2020; Üstün et al., 2021) affects the type of ANOVA analysis that is used. For example, if your research does not include a grouping factor, a two-way ANOVA (with two factors as number and difficulty) would be the right choice in your second-level analysis of the fMRI data.

***Note:*** If your study sample consisted of individuals with dyscalculia, it would be important to add the participants' overall as well as verbal IQ scores and ages as covariates to the fMRI group analysis.

16. Open the Estimate section on the SPM menu, load the SPM.mat file and run the batch.
17. To display the results of the fMRI analysis, click on the Results section on the SPM12 menu and load the SPM.mat file from the output folder.
18. To define the cluster-level threshold of statistical significance, run the Monte Carlo simulation with 10,000 iterations for the voxel-level threshold of p < 0.001 (Slotnick, 2017).
19. The result of the Monte Carlo simulation will give you the cluster-extend threshold of k ≥ voxels for getting the activations that are corrected for multiple comparisons at p < 0.05.
20. Thus, as a result of analysis of the fMRI data, you will get activations identified as significant according to the cluster-level threshold. These activations will be the results of the main effects, two-way interactions, and the three-way interaction.

### ROI analysis

**Timing: Approximately 1 h per participant for a computer (64 bit, 16 GB RAM, Intel(R) Xeon(R) CPU E5-1650 v4 @ 3.60GHz 3.60 GHz)**

21. All activation clusters for the interaction effects involving two-way and three-way interactions are defined as the region of interest (ROI). To create ROIs from activation clusters, the MarsBaR toolbox working with SPM12 is used (Brett et al., 2002). To do that:
  a. Click on the ROI definition in the MarsBaR menu.
  b. In this protocol, the ROIs are created from all clusters. To do that, click on the Get SPM cluster(s).
  c. Select the SPM.mat file.
  d. Then, display the results by accepting the default answers on the menu.
  e. On the SPM input window, click on the Write ROI(s) and select the Write all clusters.
  f. When you do this, the MarsBaR toolbox directly asks for a location to save and the program itself names the files entering the coordinates.
22. After defining ROIs, the MarsBaR toolbox is used to extract the mean percent signal change values from ROIs for each participant.
23. The mean percent signal change values of the ROIs may be used to examine significant interactions obtained from second level analysis of fMRI data .

**CRITICAL:** After extracting the mean percent signal change values of the ROIs, it would be rational not to apply further statistical analysis on the ROI data to avoid circularity. Instead, visually observe the graphed ROI data to reveal the direction of the effect.

## Limitations

Here, we present a protocol to examine the neural basis of numerosity comparison based on our previous studies examining the developmental changes in number perception and comparing children with dyscalculia and typically developing children. These two studies included children in their experimental design, so relatively low trial numbers were preferred to reduce time spent in the MRI scanner and limit head movement artifacts. Therefore, it will be important to account for the number of trials to adapt this protocol to your study.

## Troubleshooting

### Problem 1

Participants, specifically children can be anxious about MRI scanning.

### Potential solution

A Mock scanner session would be very helpful to reduce the participants’ anxiety about the MRI. Introducing scanning room and the MRI machine as well as giving fun facts would also be helpful to make children and adults be familiar with the process.

### Problem 2

Participants’ task responses cannot be recorded because the MR-compatible response pad is not working properly (step 10 in MRI scanner setup section).

### Potential solution

If you detect any equipment problems related to the response pad, firstly check the connection between the response box and the stimulus computer. Another thing to consider would be to ensure which buttons on the response pad correspond to those on the MATLAB Psychtoolbox code. If the MR-compatible pad is working properly but the response buttons are not detected by MATLAB, you can edit the paradigm code.

### Problem 3

The task displayed on the projection screen is upside down (step 2 in numerosity comparison task section).

### Potential solution

In this case, you can fix it by turning the screen upside down in the Windows operating system.

### Problem 4

When you first checked the acquired data, you discovered that the brain image has blurriness or gaps indicating a lack of data (step 7 in image acquisition section).

### Potential solution

Due to the hardware problems of the MRI machine, such as disturbances in the magnetic area homogeneity or RF (radio frequency) pulse sequence, the image can have artifacts indicating a lack of data during the scanning. In this case, evaluating the imaging performance of the MRI machine by scanning a standard imaging phantom (like plastic container filled with water) could help to understand the problems related to the machine itself.

### Problem 5

After preprocessing, when visually inspecting the data you find a misalignment of the mask (step 7 in preprocessing of the fMRI data section).

### Potential solution

In this case, you can create your own brain mask manually following the steps described in http://jpeelle.net/mri/misc/creating_explicit_mask.html.

## Resource availability

### Lead contact

Further information and requests for resources should be directed to the lead contact, Metehan ÇİÇEK (mcicek@ankara.edu.tr).

### Materials availability

This study did not generate any new materials.

## Data Availability

The MATLAB Psychtoolbox code to run the numerosity comparison paradigm during the fMRI scan is available via the Open Science Framework (https://osf.io/b85xg/) with the identifier https://doi.org/10.17605/OSF.IO/B85XG.
